# Gold Foil in Germany

**Published:** 1883-05

**Authors:** Otto Maas


### GOLD FOIL IN GERMANY.

It is only a few years since, that the gold used for filling teeth was almost entirely the products of American and English makers, and with the exception of Nedden's crystal gold, no German preparation found a ready market. It is, therefore, a pleasing sign of growing interest that Germany, within a short time, has commenced to compete with the American and English manufacturers. Mr. Wolrab, of Bremen, manufactures at present a gold filling which is not only considerable cheaper than the foreign products, but, as far as its quality is concerned, will also bear a very favorable comparison. At the meeting of the German dentists at Bremen, this gold was very favorably commented on, and an American, Prof. Truman, said that, according to his own experience, Mr. Wolrab's gold was equal to the best of American preparations.—OTTO MAAS, in "*Die Zahntechnische Reform.*"

